# Password Security as a Game of Entropies

**DOI:** 10.3390/e20050312

**Published:** 2018-04-25

**Authors:** Stefan Rass, Sandra König

**Affiliations:** 1 System Security Group, Institute of Applied Informatics, Universität Klagenfurt, 9020 Klagenfurt, Austria; 2 Austrian Institute of Technology, Center for Digital Safety & Security, 1210 Vienna, Austria

**Keywords:** game theory, security, entropy, passwords

## Abstract

We consider a formal model of password security, in which two actors engage in a competition of optimal password choice against potential attacks. The proposed model is a multi-objective two-person game. Player 1 seeks an optimal password choice policy, optimizing matters of memorability of the password (measured by Shannon entropy), opposed to the difficulty for player 2 of guessing it (measured by min-entropy), and the cognitive efforts of player 1 tied to changing the password (measured by relative entropy, i.e., Kullback–Leibler divergence). The model and contribution are thus twofold: (i) it applies multi-objective game theory to the password security problem; and (ii) it introduces different concepts of entropy to measure the quality of a password choice process under different angles (and not a given password itself, since this cannot be quality-assessed in terms of entropy). We illustrate our approach with an example from everyday life, namely we analyze the password choices of employees.

## 1. Introduction

A sophisticated (cyber-) attack on a company often involves some sort of information gathering in the beginning. The desired information may include knowledge about the premises but also about the internal processes. This situation can be considered as a competition between the attacker, who wants to gain information, and a company trying to make acquiring this knowledge as hard as possible. Games constitute a natural model for this kind of conflict. Many games in cyberwarfare are about information. The quantitative treatment of information by concepts of entropy lets us define games about information losses and gains, by letting the competition reward either player in terms of entropies.

The uncertainty in such a model is not only about which game is played but also about the adversary’s incentives. Adversary modeling is a common phase in the conceptualization of security systems. In game-theoretic security models, however, the class of zero-sum games offers an appealing degree of freedom, since it is easy to show that a zero-sum regime yields the minimal rewards independently of the adversary’s payoff structure. That is, assuming that the adversary’s intentions are exactly opposite to the defender’s assets, i.e., the attacker seeks to cause maximal harm, any other incentive of the attacker can only improve the defender’s situation [1]. The application of game theory to model attacker-defender scenarios has been studied in a considerable body of literature, among them network defense and general resource allocation games [2,3,4,5,6], to name a few.

For this reason, we propose zero-sum games as models for the information gain competition, which specifically is about passwords. Even though passwords are the weakest form of authentication, they are nonetheless among the most common entry gates to any system, and usually, grant access to much stronger cryptographic assets like keys. So requiring a high entropy (or bitlength) for a cryptographic key does not help much if its usage is guarded by a password that is easy to guess.

Passwords are technically simple but tricky to handle practically, since the (cognitive) efforts to memorize a password compete with those to guess it. In other words, people may prefer simple passwords to memorize them easily, but then accept an increased risk of those to be guessed (or to appear in lists of common passwords on the Internet). Password policies play a twofold role herein (1) enforcing a closer-to-uniform distribution of the password generation output, and (2) harden a brute-force guessing of the password. Typically, such policies prescribe a frequent change of the password, but towards easing matters of memorizing the next password, people may keep the difference between the old and the new password as small as possible (say, by adding a counter at the end of the password, or by just switching two letters).

Capturing this in a game-theoretic fashion is not obvious since we use entropies to measure the quality of the password choice policy, but the effect of “ineffective changes” to a password (following the prescriptions of the policy) add costs to the game that appear *in-between* the repetitions and not during the game-play itself. This means that standard static games are not directly suitable (neither are dynamic games, since they mainly change the payoff structure between repetitions, but do so disregarding any cost for the switch). Section 1.1 below is thus partly devoted to the description of an extended static (matrix) game model that lets us account for these “strategy switching costs”.

This work proposes a model to optimize the tradeoff between the conflicting requirements of password choices, basically between low memorability and difficulty of guessing. Either can be supported by admitting or forbidding certain choices of passwords, and game theory can be used to find an optimal mix of strict policies and free choices for passwords. Suppose that several password choice policies exist, then besides changing the password, why not let us change the password policy too? For example, if a policy is too strict, then people will find ways to ease their life with the password, say, by storing the password in an unsafe place. The idea of this work is a random change between password policies upon each (enforced) change of a password itself. Once a password has expired, the next choice may be subject to a much less restrictive choice, thus adding convenience for the user for the time of validity of the next password (e.g., it may—temporarily—be a simple password that is easy to memorize). In the next round, the password policy then again randomly changes to prescribe a much harder to guess the password, that is harder to memorize. So, we have randomly alternating features of the difficulty of guessing vs. ease of memorizing, and game theory helps to find the optimal tradeoff. This is what the rest of the paper will be about: we start with related work on password quality measures (as we need them) in Section 1.1, devoting the remainder of Section 1 to outlining the contributions in Section 1.2 and reviewing basic game theoretic terms as needed. The model is formally introduced in Section 2, and applied in Section 3 to the policy of a password protecting an employee’s PC. Discussion and concluding thoughts follow in Section 4.

### 1.1. Related Work

Entropy is commonly used as a measure of password quality, where Shannon entropy is the most popular measure [7], besides related notions like guessing entropy or min-entropy [8,9]. A recognized misperception [10] is that entropy is not a measure of a password itself but rather a rating of the password generation process. This process is typically subject to a variety of constraints, known as password policies [10,11]. Technically, their purpose is to strengthen the choice process towards the maximal achievable complexity of guessing attacks. This complexity can itself be taken as an alternative measure of password strength [7,8,12], besides entropy. Any such brute-force-attack based measure can at best be an upper bound to the quality of a password since the possibility of a better dictionary or more efficient guess generator remains in any case. In this regard, [8] provides an excellent discussion of the issues with such an approach. A discussion of entropy vs. password complexity is given by [13]. The use of entropy is, however, no less tricky in general, since high Shannon-entropy (usually interpreted as high uncertainty) is not necessarily implying high efforts to guess the password (we give an explicit example later).

Password policies increase the strength of a password (no matter if computed in terms of entropy or complexity of password cracking), at the cost of adding cognitive workload for the user; in the worst case, ending up with dramatically decreased usability. The unpleasant implication can be that passwords may be written down somewhere, or otherwise need to be stored in a safe place (in many cases protected by a master password). Password storage is thus an independent issue with lots of software solutions, often based on cryptographic notions, e.g., [14]. Alternative kinds of passwords that explicitly address issues of social engineering are based on graphical and geometric challenges to authenticate [15,16,17]. Though our model, in theory, could be applied to such mechanisms too, we do not explore that route in this work.

The exploration of the tradeoff between password resistance and usability is not new [18], and previous investigations primarily make use of optimal choice rules to optimize this tradeoff in light of different assumptions on the adversary. We adopt such assumptions too, but for the first time and thus contrary to past related work, we add the element of efforts to switch passwords rather than to just choose them. Changing one password into a literally “close” new one may play into the adversary’s hands; for example, if the policy prescribes to not use the last 10 passwords, then a simple way of satisfying this rule is appending a counter to the password. However, if the adversary has already guessed the current password, the next guess will be considerably more likely to succeed if the switch from the current to the next password was made with minimal (cognitive) efforts. This detail is not trivial to include in a game-theoretic model but can be accounted for, as we show in Section 2.

### 1.2. Contribution

With lots of empirical work having been done on understanding what passwords typically look like in real life (see [11,19,20] and references therein), the question about the password change process has received considerably less attention [21]. Many different password policies and design principles for policies and passwords exist, which are exclusively based on plausible intuitions and for a tradeoff between usability and password strength.

The goal of this work is a formalization of the choice process, by combining different password policies into a *randomized* password policy, which is provably optimal based on the underlying set of input policies. We apply two-player games for that matter, where the first player’s duty is creating a password from a randomly prescribed policy (the random prescription is what we will optimize). The second player’s goal is cracking the password by any means. As an implicit assumption on the adversary, we shall assume that the password change intervals are such that the password search space as implied by the password policy is so large that it cannot be all searched within the validity period of the current password. For “unrestricted policies”, i.e., passwords chosen as words from the native language, the search space would be very small (a few hundred thousand words), so that the validity period would almost vanish. In such cases, we would need a lockout counter upon incorrect passwords in a realistic setting, so that the maximum failure count would bound the number of trials. In more restrictive policies, such as if the password is a string containing at least some upper- and lower-case letters, special characters, etc., the validity period can be prolonged accordingly (though a lockout counter is nonetheless advisable but admitting more failures than in the previous case). For simplicity, we shall bear such considerations in mind in the following, but not explicitly refer to them. Specifically, we will not use the lockout counter assumption in the numeric examples to avoid trivialities (and tedious probabilistic arguments giving the payoffs in our game via the likelihood to guess the correct password from several hundred thousand candidates within a few trials).

The more interesting detail about applying game theory here is that password changes are often made dependent on past passwords (simply to minimize the cognitive efforts to memorize the new password based on the known old one). This adds a dimension of payoff *between* repetitions of the game, which is not part of standard game-theoretic considerations. Thus, the game model applied here involves an additional measure of complexity in the change of the password, additionally to the quality measures for the password choice itself. This leads to the problem of quantifying the “mixedness” of equilibrium strategies in the two-person game, in favor of “less mixed” (i.e., “more pure”) strategies over others. One work doing this in the security context is [22], who use relative entropy (Kullback-Leibler divergence) to favor pure strategies over randomized ones. A different, and more general approach that we shall use below too is that in [23] based on [24], who allow for two kinds of payoffs: one for the game-play itself, and an independent one to capture the cost of switching from one strategy to another in the next repetition of the game. We shall apply this idea to password choices, letting the payoff in the game be the chance to crack the password, relative to the investment of player 1 to choose it (measured by entropy). The cost to change the password will, similar as in [22], be measured by the relative entropy of the new information token (the new password) in light of the previously known information (the old password).

### 1.3. Preliminaries

Hereafter, we shall consider multi-objective finite two-player games, which we denote by sets of payoff matrices $A_{1},\ldots ,A_{d}\in R^{n\times m}$ for a set of *d* (perhaps interdependent) goals 1,…,d. Player 1 will be the defender, being the entity that chooses its passwords according to a set of *n* different password policies. The strategy space for the first player will thus be a set of password policies from which a new one is prescribed whenever the password needs to be changed. The optimal choice as a mixed strategy will be derived from the game, and can as such be thought of as a “meta-policy”, in the sense that the game randomly (yet optimally) prescribes which password rules to apply upon a change (we will revisit this aspect explicitly in the concluding Section 4). While the equilibrium prescribes the frequencies of each pure strategy to be played, it does not give explicit instructions on the order of choosing them.

Player 2 is the attacker, having a list of *m* actions to choose from, each of which is another (heuristic) search strategy on the password space or general password cracking approach (e.g., using rainbow tables, resetting lockout counters, etc.). Let us take $PS_{1},PS_{2}$ as sets of pure strategies with cardinalities $\left|PS_{1}\right|=n$ and $\left|PS_{2}\right|=m$. Furthermore, we write S(M) for the set of mixed strategies (the simplex) over the finite set *X*; mostly, we will have $M=PS_{1}$ or $M=PS_{2}$. The payoffs in the mixed extension of the game are vector-valued functions $u_{i}:S\left(PS_{1}\right)\times S\left(PS_{2}\right)\to R^{d}$ for d≥1. Since the game is multi-objective, we look for Pareto-Nash equilibria $\left(x^{*},y^{*}\right)\in S\left(PS_{1}\right)\times S\left(PS_{2}\right)$, which for *minimizing* players, satisfies

$$\begin{array}{cc} u_{1}\left(x^{*},y^{*}\right) & \leq_{1}u_{1}\left(x,y^{*}\right)\;\forall x\in S\left(PS_{1}\right),\\ u_{2}\left(x^{*},y^{*}\right) & \leq_{1}u_{2}\left(x^{*},y\right)\;\forall y\in S\left(PS_{2}\right), \end{array}$$

where $a\leq_{1}b$ holds if and only if at least one coordinate in a is ≤ than the respective coordinate in b. In more practical terms this means that changing the strategy makes sense if we improve in at least one respect, i.e., at least one component of the utility function gets reduced. Intuitively, a Pareto-Nash equilibrium is thus such that any unilateral deviation from the optimum $\left(x^{*},y^{*}\right)$ will worsen the revenue for the deviating player in at least one of its goals. (Note that both the $\leq_{1}$-relation and its complement set, the element-wise ≥ relation between vectors, are only partial orders on $R^{d}$).

The computation of Pareto-Nash equilibria can be reduced to the computation of (standard) Nash equilibria, as shown by [25], and shall not burden us much further in the following. Nonetheless, we will revisit the computational matters at the end of the next section, where the particularities of password *changes* need to go into the equilibrium computation.

Since we will make simultaneous use of different entropies, we use Rényi-Entropy as a unifying concept, where the *s*-order Rényi-entropy for a random variable *X* with a discrete probability distribution $p=\left(p_{1},\ldots ,p_{n}\right)\in S\left(\left\{1,2,\ldots ,n\right\}\right)$ is defined for s>0 as

(1) $$H_{s}\left(X\right)=\frac{1}{1-s}\log_{2}\left(\sum_{i=1}^{n}p_{i}^{s}\right).$$

As a measure of distance between the distributions p,q of random variables X,Y, later helping us to quantify the effort for changing passwords, we will use the Kullback–Leibler (KL) divergence, being

$$KL\left(X∥Y\right)=\sum_{i}p_{i}\cdot \log\frac{p_{i}}{q_{i}}.$$

## 2. Model

In the following, we consider a two-player zero-sum game in normal form where both attacker and defender have a finite set of strategies denoted by $a_{i}$ for the attacker and $d_{j}$ for the defender, respectively. In a situation where the attacker wants to disclose a ‘secret’ *X*, the defender aims to make this as hard as possible. Thus, we define a game with payoffs

$$u_{ij}=H\left(X|d_{i},a_{j}\right),$$

where *H* denotes the entropy of the random variable *X*. The zero-sum assumption takes into account that the intentions of attacker and defender are opposite, i.e., the attacker wants to minimize the uncertainty about *X* while the defender wants to maximize it.

Up to this point, the actual game-play is still an oversimplification of the real matters of information games in security. For a password, we typically have opposing goals being (i) hardness to guess it, and (ii) easiness to memorize. Obviously, the two conflict, but more importantly, quantifying the two requires different concepts of entropy. Let *X* be the random variable from which we sample the password. The effort of guessing a random password *x* is measured by the min-entropy $H_{\infty }\left(X\right)$. On the contrary, the average lot of bits to encode (store) the password is measured by the Shannon entropy $H_{1}\left(X\right)$, where $H_{s}$ is the Rényi-entropy of order *s* here; see Equation (1). It is well known how to construct random variables with constant min-entropy but arbitrarily large Shannon-entropy: Let 0<q<1 be arbitrary but fixed. For every n∈N, define a random variable $X_{n}$ over $\left\{0,1,\ldots ,n\right\}$, whose distribution puts the mass of 0<q<1 on the outcome $X_{n}=0$, and uniformly assigns the mass (1−q)/n to all outcomes $X_{n}>0$. Then, letting n→∞, it is easy to see that the best guess is $X_{n}=0$ with a constant chance of winning (reflected by the min-entropy being $H_{\infty }\left(X_{n}\right)=-\log_{2}q$ for all *n*), although the Shannon-entropy grows to infinity as $H_{1}\left(X_{n}\right)\in \Omega \left(\log n\right)$.).

Since the two goals are opposing, the above game is at least two-dimensional, where we seek to maximize the min-entropy and minimize the Shannon entropy. As a natural constraint, we have $H_{\infty }\left(X\right)\leq H_{1}\left(X\right)$. However, this is not even the end of the story since passwords need to be changed on a regular basis. As a simplification of the game model, let us assume that the change is done upon every repetition of the game (exploiting the static but repeated nature of the game here). Then, users tend to choose passwords that are close to their previous ones or familiar for other reasons (say, being a word from the native language). In general, if $p_{t}$ is the password at time step t∈N, then the preferred next choice of a password $p_{t+1}$ should be (i) still hard to guess (high min-entropy); (ii) also easy to memorize (low Shannon entropy); and (iii) for the previous sake, not too different from the past choice $p_{t}$.

This induces a third aspect to consider as a payment made upon changing the strategy between the *t*-th and (t+1)-th repetition of the game. In fact, the change from the current to the next strategy is tied to a payment made for that change. Obviously, not changing the password choice rule at all is the easiest, but a good choice should also change the way in which the new password is chosen so that the information about the current password is invalidated for guessing the new password. For example, if the adversary gathers information from social media to help it guess the current password of a user under the hypothesis that the choice is made close to the data in the adversary’s possession (e.g., pets names, favorite artist name important dates, etc.). So, the new choice should be made on different grounds. Such a change in the password choice strategy is, however, cognitively more involved than just staying with the past choice strategy. In the latter case, the new password would merely be another pet’s name, another favored artist or another important date.

Heuristically, let us suppose that *X* is the past random variable (distribution) from which $p_{t}$ was sampled and that the next choice rule is the random variable *Y*. The cost of switching from *X* to *Y* can be measured by the Kullback-Leibler divergence KL(Y∥X), which—intuitively—quantifies how much more information is required to encode a sample from *Y* when the (past) encoding of *X* is used (corresponding to the cognitive “inertia” that makes us apply our past thinking (*X*) to the future (*Y*)). If the distribution from which the password is chosen is the same between two instances, then we may use the information gain or other measures to quantify this “cost”.

To put this to work in a game, let us presume that there are a few (finitely many) different ways known to the user in which passwords can be chosen. Mostly, these are the usual heuristics according to which passwords can (and should) be formed. These different ways make up the strategies in the game, besides others that come with the password management process as such (in Section 3, we will mention some of these actions).

This makes it a total of three payoffs to simultaneously optimize in our data collection game for password authentication:

- Maximization of min-entropy
- Minimization of Shannon-entropy
- Minimization of the efforts to switch passwords.

The third point is that the cost for switching is incurred by the cognitive effort to memorize the new password over the old one. Expressed in terms of Kullback-Leibler divergence, this measures how much more information we need to memorize for the new password *y*, sampled from the random variable (=policy) *Y*, when the previous password *x* was sampled from the random variable (=policy) *X*. The switching cost is thus the amount of information KL(Y∥X) required to memorize *y* when *x* is already known. In particular, this cost is low if the new password is similar to the old one, e.g., constructed by adding a number at the end of the old one. Still it fits awkwardly into a classical game-theoretic setting, since these costs occur *between* the repetitions of the game, as opposed to the other two that occur when the *current instance* of the game *terminates*. A formal inclusion of these switching costs is, however, not difficult [26].

### Games with Switching Cost for Mixed Strategies

Since only player 1 is concerned with efforts for choosing a new password relative to the existing one, we will only include these costs in the equilibrium computation. Intuitively, if the current strategy is $i\in PS_{1}$ and the next strategy (chosen at random according to the equilibrium distribution $x^{*}\in S\left(PS_{1}\right)$) is $j\in PS_{1}$, then let us define a *switching cost* $s_{ij}$ for this action. This cost appears *between* two independent repetitions of the game and reduces the payoff in the next round accordingly. Since this cost may be different in each round where *j* is played, we cannot plainly include it in the payoff structure. In [4], the matrix game model was generalized towards including an additional (n×n)-cost matrix $S={\left(s_{ij}\right)}_{i,j=1}^{n}$ over $PS_{1}$. Player 1 has this as an additional goal to minimize, and for computing a zero-sum equilibrium $x^{*}$, needs to solve the following nonlinear program for an arbitrary α>0 and an auxiliary variable *v* (in a single-objective game, *v* would be the game’s saddle-point value $v=x^{*}\cdot Ay^{*}$. Since the method to solve the game here is based on a scalarization of the multi-objective into a single-objective game, *v* plays the same role as in the linear program for a classical game but loses the interpretation as a saddle-point value. Hence we refrain from referring to it like this, and refer to it as a mere “auxiliary variable”.)

(2) $$\left.\begin{array}{cccc} \mathrm{minimize} & v & & \mathrm{over}\;x\in S(PS_{1})\\ \mathrm{subject}\;\mathrm{to} & v & \geq & \alpha \cdot x^{T}Sx+\left(1-\alpha \right)x^{T}Ae_{i}\;\mathrm{for}\;i=1,\ldots ,m;\\ & \sum_{j=1}^{n}x_{j} & = & 1;\\ & x_{j} & \geq & 0,\;\mathrm{for}\;i=1,\ldots ,n. \end{array}\right\}$$

This program assumes a minimization of all goals, and a single payoff structure $A\in R^{n\times m}$. Even without the switching cost, our game is multi-objective which we solve by applying the method of [25]. This method basically scalarizes the vector-valued payoffs into $A=\alpha_{1}\cdot A_{1}+\alpha_{2}\cdot A_{2}+\ldots +\alpha_{d}\cdot A_{d}$ for arbitrarily chosen values $\alpha_{1},\ldots ,\alpha_{d}>0$ and $\alpha_{1}+\ldots +\alpha_{d}=1-\alpha$ (remember that the switching cost goes into this scalarization as the (d+1)-st goal, which is why the weights $\alpha_{i}$ only sum up to 1−α here).

A closer look reveals that (2) is almost the familiar linear program known from basic game theory, and the switching cost just added the quadratic (and only nonlinear) term to the constraints.

The coefficients $\alpha ,\alpha_{1},\ldots ,\alpha_{d}$ are admittedly arbitrarily chosen (under the given constraints), but their choice determines which equilibrium is found as the solution [25]. These can be interpreted as *individual importance* values assigned to different goals. For the practical example to follow at the end of Section 3, we will show how different settings deliver different outputs (somewhat more technically, the choice determines which point on the Pareto-front is found by the optimization).

The equilibria in the so-generalized games are as non-unique as their classical counterparts (after all, the computation reduces to finding standard Nash equilibria [25], each of which delivers another valid equilibrium in our game with the switching cost). Indeed, multiple different equilibria may be obtained by changing the α-coefficients above [25]. Our experimental evaluation of (2) showed, however, that the program seems to be ill-conditioned (or at least numerically involved) in general.

## 3. Example

We illustrate our approach by considering a small example from everyday life where an attack involves guessing a password on an employee’s PC. While in theory, all possible passwords are equally likely to be used, this is often not the case in practice. People tend to use simple passwords that are easy to memorize or even if a randomly generated password is used it might be available in written form at the workplace. The attacker now looks for ways to get some information that helps him reduce the uncertainty about the password while the defender tries to keep the maximal uncertainty induced by the uniform distribution over all potential passwords.

The list of potential strategies for the attacker, which may (among others) include:

1. trials of standard passwords
2. social engineering
3. checking publicly available personal information (obtainable from social media)

The full collection of the attacker’s actions makes up player 2’s strategy set in the game. For illustrative sake, let us confine ourselves to the three example strategies above.

Player 1 is the attacker’s victim, who is supposed to choose a password that is easy to memorize, hard to guess and efficient to change (without much cognitive workload for memorizing the new password). The game for player 1 is a matter of choosing the best password choice strategy. To simplify matters here, let us consider the following options as “defense actions” in the game:

1. Adhere to a password policy, say, the password should have at least 8 letters and must contain at least one upper-case, one lower-case letter, one digit and one special character (from a total of 10 such permitted characters).
2. Choose the password freely, but with at least 9 characters.

Neither strategy is obviously better, since guessing a password is certainly harder following the policy (the first strategy), but at the same time, memorizing that password is more difficult. Likewise, choosing a password unrestrictedly lets us easily memorize it (More sophisticated heuristics of password choices as derived from an English sentence are not considered here. However, we stress that even a free choice *may* adhere to a policy when letters are replaced by similar-looking special characters or numbers.).

We are now ready to define the payoffs in the game, based on known entropy estimates regarding the English language. Since most resources refer to Shannon entropy in this regard, our example choice for min-entropy and Kullback-Leibler divergences will rely on distributional assumptions.

*Choice rule $X_{1}$:* Using the law of inclusion-exclusion, we find that ≈$3.1\times 10^{14}$ passwords are admissible, giving an equal Shannon- and min-entropy of $H_{1}=H_{\infty }\approx 48.14$ bit. Dividing this number by 8, we end up with the per-letter entropy of ≈6.02 bits. Again, to keep the example simple and to easily compare the two strategies, let us assume that the average human may prefer passwords that are similar to at least some English words so that they can memorize it easily. Thus, among the total of $3.1\times 10^{14}$ words, let us restrict the practical choices to a set whose size equals the English vocabulary, i.e., 171,476 passwords (note that this at the same time resembles the perfectly uniform choice over the English language, at least the distribution shape being what the password policy should ultimately enforce). The Shannon- and Min-entropies, in that case, are equal as $H_{1}=H_{\infty }\approx 17.39$ bits.

*Choice rule $X_{2}$:* Now let us consider the *fully unrestricted choice* of a password: Based on [27] (following the earlier work of [28]), the entropy rate of English is 1.58 bits per character. Thus, choosing from the purported total of 171,476 words in current use in English [29], approximately 3000 of which suffice to handle the daily business [30]. The maximum entropy for *n* letter words has in [27] been found between 8.31 and 11.43 characters (hence the prescription in the above choice rule). Thus, the average Shannon-entropy is ≈9×1.58=14.22 bits.

For the min-entropy, let us assume that choosing core words is 10-times more likely than choosing words from the rest of the English vocabulary, and let either choice be uniform (again, for simplicity only) in lack of empirical min-entropy estimates (hose are seemingly only available for random number generators [31], but appear rare for spoken or written natural language). Under this assumption, the 3000 core words take up a fraction of 10/11 of the overall mass, leaving a fraction of 1/11 to the remaining words. We end up with the mass assigned to the 3000 core words, whose negative logarithm is the min-entropy, i.e., $H_{\infty }=-\log_{2}\left(\frac{10}{11}\frac{1}{3000}\right)\approx 11.69$ bits.

Cost of Password Changes:

The latter assumption simplifies matters of computing (estimating) the Kullback-Leibler divergence between the strategies. We shall use R [32] and the entropy package therein [33], taking the masses:

*Policy-based choice:*$f_{1}\left(x\right)=1/\left(171\;476\right)$ for all *x*.

*Free choice:* $f_{2}\left(x\right)=\left\{\begin{array}{cc} \frac{10}{11}\cdot \frac{1}{3000}, & \mathrm{for}\;1\leq x\leq 3000,\\ \frac{1}{11}\cdot \frac{1}{168\;476}, & \mathrm{otherwise}. \end{array}\right.$

Under this setting, the respective KL-divergences come to $KL(f_{1}∥f_{2})\approx 3.29$ and $KL(f_{2}∥f_{1})\approx 4.86$. Intuitively, this makes sense, since it appears more difficult to switch from a free choice to a restricted one than from the opposite. When the choice remains according to the policy, suppose that the entropy gain is determined by at least one letter being changed relative to the last password. For a free choice, we would get the per-letter entropy rate [27] of 1.58. For the policy-based choice, we get $1/8\cdot \log_{2}\left(1/171\;476\right)\approx 2.17$.

This completes the cost matrix for switching between the policies as

$$\overset{\begin{array}{cc} \;X_{1}\; & \;X_{2} \end{array}}{\begin{array}{cc} \begin{array}{c} X_{1}\\ X_{2} \end{array} & \left(\begin{array}{cc} \mathrm{entropy}\;\mathrm{rate}\;\mathrm{for}\;X_{1} & KL(f_{1}∥f_{2})\\ KL(f_{2}∥f_{1}) & \mathrm{entropy}\;\mathrm{rate}\;\mathrm{for}\;X_{2} \end{array}\right) \end{array}}=(\begin{array}{cc} 2.17 & 3.27\\ 4.86 & 1.58 \end{array})$$

### Payoffs for Password Choices

To avoid trivialities by the humble fact that a brute-force trial of all passwords will always succeed, it appears reasonable to assume that this attack will succeed for a free choice of a password, but fail against a policy-compliant choice. Suppose that social engineering, depending on the awareness, may be useful to dig up the password with a likelihood of, say 0.5. Likewise, and also depending on the awareness, suppose that a password is written down with a likelihood of 0.7 for a policy based choice, but only with a likelihood of 0.2 for a free choice. In that case, we can set the likelihoods for a successful attack to be

$$P=\overset{\begin{array}{ccc} \;\begin{array}{c} \mathrm{standard}\\ \mathrm{passwords} \end{array} & \begin{array}{c} \mathrm{social}\\ \mathrm{engineering} \end{array} & \begin{array}{c} \mathrm{check}\\ \mathrm{notes} \end{array} \end{array}}{\begin{array}{cc} \begin{array}{c} \mathrm{choice}\;\mathrm{rule}\;X_{1}\\ \mathrm{choice}\;\mathrm{rule}\;X_{1} \end{array} & \left(\begin{array}{ccc} \;0\; & 0.5\; & 0.7\\ \;1\; & 0.5\; & 0.2 \end{array}\right) \end{array}}$$

Remember that the overall objective of the password choice game is maximizing the utility of player 1, who chooses the password. Since this person is typically unaware of the actual incentive that an adversary has, we cannot make reliable assumptions on the payoffs of the attacker. In the absence of this information, the simplest way is setting the adversary’s payoffs to the negative values of player 1’s revenues. The respective revenues for player 1 are:

**Rememberability** (Shannon entropy):

$$R=\overset{\begin{array}{ccc} \;\begin{array}{c} \mathrm{standard}\\ \mathrm{passwords} \end{array} & \begin{array}{c} \mathrm{social}\\ \mathrm{engineering} \end{array} & \begin{array}{c} \mathrm{check}\\ \mathrm{notes} \end{array} \end{array}}{\begin{array}{cc} \begin{array}{c} \mathrm{choice}\;\mathrm{rule}\;X_{1}\\ \mathrm{choice}\;\mathrm{rule}\;X_{1} \end{array} & \left(\begin{array}{ccc} \;17.39\; & 17.39\; & 17.39\\ \;14.22\; & 14.22\; & 14.22 \end{array}\right) \end{array}}$$

**Guessing** (min-entropy):

$$G=\overset{\begin{array}{ccc} \;\begin{array}{c} \mathrm{standard}\\ \mathrm{passwords} \end{array} & \begin{array}{c} \mathrm{social}\\ \mathrm{engineering} \end{array} & \begin{array}{c} \mathrm{check}\\ \mathrm{notes} \end{array} \end{array}}{\begin{array}{cc} \begin{array}{c} \mathrm{choice}\;\mathrm{rule}\;X_{1}\\ \mathrm{choice}\;\mathrm{rule}\;X_{1} \end{array} & \left(\begin{array}{ccc} \;17.39\; & 17.39\; & 17.39\\ \;11.69\; & 11.69\; & 11.69 \end{array}\right) \end{array}}$$

Since attacks have a probabilistic chance to succeed, the expected payoffs are given by the element-wise (Hadamard) products

$$A_{1}=P\circ R=\left(\begin{array}{ccc} 0 & 8.695 & 12.173\\ 14.22 & 7.11 & 2.844 \end{array}\right)\;\mathrm{and}\;A_{2}=P\circ G=\left(\begin{array}{ccc} 0 & 8.695 & 12.173\\ 11.69 & 5.845 & 2.338 \end{array}\right)$$

Solving the game according to the method of [23] with the nonlinear program (2), the final optimal choice rule is a mix between policy-based selection (≈72.43%) and free choices (≈27.57%). That is, in roughly one out of four cases we should choose freely (i.e., apply $X_{1}$) while we should comply with the policy in the remaining three out of four cases (i.e., apply $C_{2}$). Note that the saddle point value of the game (here ≈2.23) enjoys no direct interpretation (not even as an entropy) since it arises as a weighted sum of Shannon-, min-entropy, entropy rate and KL-divergences. The important insight from the game is thus the recommendation for the optimal password choice rules. This result has been obtained while assigning 60% importance to matters of memorability, 20% weight for the difficulty of guessing, and another 20% relevance (importance) for the switching cost.

Changing these priorities, say 10% for memorability, 70% for the difficulty of guessing, and 20% for the cost of changing the password alters the answer: now, the optimal password choice rule would be a mix of policy-based choices (≈41.25%) and free choices (≈58.74%).

To conclude the interpretation of results, note that the optimal value *v* from (2) has no meaning by itself, but given the optimal $x^{*}$, we can compute bounds to the entropies in the worst-case: if player 1 plays $x^{*}$, then player 2 has the payoff structure ${\left(x^{*}\right)}^{T}\cdot A_{j}\in R^{d}$ for j=1,2,…,d. Since this is a vector, and player 2 is maximizing (i.e., minimizing the revenue of player 1), it is a trivial matter of finding the individual worst-case strategies and bounds for all goals (though only the min-entropy bound may be of real interest for the attacker, since memorability of the password for player 1 is none of its concerns.):

**Worst-case strategy for player 2:** this is $\mathrm{argmax}\{{\left(x^{*}\right)}^{T}\cdot A_{j}\}$.

**Worst-case payoff for the *j*-th goal:**$v_{j}=\max\{{\left(x^{*}\right)}^{T}\cdot A_{j}\}$. This value is an *upper bound* (since player 1 is minimizing) for all that player 1 can suffer by player 2’s actions. Moreover, by the definition of a Pareto-Nash equilibrium, player 1 cannot uniformly improve its situation by any alternative strategy $x\neq x^{*}$ (see [1] for a more comprehensive treatment and proofs in the classical setting without switching cost).

Evaluating $v_{j}$ for the min-entropy goal thus yields an upper bound for the difficulty of guessing the password. Computing $v_{j}$ for the Shannon entropy goal respectively shows the worst-case information to be memorized (on average), when playing against the attacker.

## 4. Discussion

The model and ideas presented in this work should be taken as a kind of “position statement”, in recognition of the oversimplification of the cognitive issues that determine password choice processes. As such, the practical instantiation of the model must rest on stronger empirical data and knowledge about how passwords are chosen, memorized and regarding the awareness of guessing and other attacks, such as reported in [11,19,20,21], to name a few. Nevertheless, we believe that entropies of a proper form can provide a sound information-theoretic measure in a game-theoretic treatment of password security. In general, game theory has proven to be a powerful and natural tool in security (see [2,34] to mention only two resources). Password security is a particularly challenging issue here, not on the technical level, but on the level of cognitive processes that determine how individuals act (optimally in their own perception). So, the lesson taught by our exposition relates to the need for different sorts of entropies to capture the conflicting goals of password choices. The human inertia of changing already memorized data shows that classical games do not directly apply here, but can be suitably adapted to fit the problem.

Unlike many other games, however, the practical meaning of mixed strategies is easy here: whenever the password is to be changed, the system may choose a policy for the password at random according to the equilibrium distribution $x^{*}$. Having a “free choice” (as we used above) among the set $PS_{1}$ adds the incentive for the user of occasionally being *allowed* to choose a password from the native language, being burdened only part-time with a more complex policy. Since we assumed a zero-sum competition, the resulting security in terms of entropies, regarding memorability and difficulty of guessing, remains assured and optimal in a Pareto-sense, regardless of the true incentive of the attacker [1].

The main goal beyond showing how password security can be viewed as a game of entropies is to stimulate further research in this direction, primarily from the psychological and cognitive domain, but also related to theoretical matters of entropy estimation. For Shannon-entropy, the problem has received much attention [35], but this is not the case for min-entropy. Maybe password games can be a starting point for fruitful research in both areas.
